# The impact of a cancer diagnosis on weight change: findings from prospective, population-based cohorts in the UK and the US

**DOI:** 10.1186/1471-2407-14-926

**Published:** 2014-12-09

**Authors:** Sarah E Jackson, Kate Williams, Andrew Steptoe, Jane Wardle

**Affiliations:** Health Behaviour Research Centre, Department of Epidemiology and Public Health, University College London, London, UK; Psychobiology Group, Department of Epidemiology and Public Health, University College London, London, UK

**Keywords:** Weight loss, Body weight changes, Cancer diagnosis, Overweight, Obese, Cancer survivors

## Abstract

**Background:**

Obesity is a risk factor for cancer incidence and survival, but data on patterns of weight change in cancer survivors are scarce and few stratify by pre-diagnosis weight status. In two population-based cohorts of older adults, we examined weight change in cancer survivors and cancer-free controls in relation to baseline weight status.

**Methods:**

In the English Longitudinal Study of Ageing (ELSA) and the Health and Retirement Study (HRS), we identified participants diagnosed with cancer who had pre- and post-diagnosis BMI data (ELSA *n* = 264; HRS *n* = 2553), and cancer-free controls (ELSA *n* = 1538; HRS *n* = 4946). Repeated-measures ANOVAs tested three-way interactions by group (cancer/control), time (pre-/post-diagnosis), and pre-diagnosis weight status (normal-weight/overweight/obese).

**Results:**

Mean BMI change was -0.07 (SD = 2.22) in cancer survivors vs. +0.14 (SD = 1.11) in cancer-free controls in ELSA, and -0.20 (SD = 2.84) vs. +0.11 (SD = 0.93) respectively in HRS. Three-way interactions were significant in both cohorts (ELSA *p* = .015; HRS *p* < .001). In ELSA, mean BMI change in normal-weight cancer survivors was +0.19 (SD = 1.53) compared with -0.33 (SD = 3.04) in obese survivors. In ELSA controls, the respective figures were +0.09 (SD = 0.81) and +0.16 (SD = 1.50). In HRS, mean change in normal-weight cancer survivors was +0.07 (SD = 2.30) compared with -0.72 (SD = 3.53) in obese survivors. In HRS controls, the respective figures were +0.003 (SD = 0.66) and +0.27 (SD = 1.27).

**Conclusion:**

Over a four-year period, in two cohorts of older adults, cancer survivors lost weight relative to cancer-free controls. However, cancer survivors who were obese pre-diagnosis were more likely to lose weight than healthy-weight survivors or obese adults without a cancer diagnosis. Whether this was due to differences in clinical status or deliberate lifestyle change triggered by the cancer diagnosis is not known. Further research is needed to establish why weight loss occurs more frequently in cancer survivors who were obese at diagnosis, and whether this has favourable effects on mortality.

**Electronic supplementary material:**

The online version of this article (doi:10.1186/1471-2407-14-926) contains supplementary material, which is available to authorized users.

## Background

There is growing interest in the role of body weight in cancer, both in terms of its effect on incidence and on survival. Overweight and obesity are associated with increased risk of a number of the most common cancers [1, 2]. A growing body of evidence also identifies obesity as a risk factor for recurrence of the primary cancer, second primary cancers, reduced treatment effectiveness, treatment-related complications, and mortality [3–11].

Although a number of studies have described changes in weight and other anthropometric markers in cancer patient populations [12–15], the majority do not compare changes to cancer-free controls, making it impossible to determine whether the changes reported are related to the cancer diagnosis or reflect typical changes over time. Two exceptions are the Norwegian Women and Cancer study, which found BMI change over a six-year period from pre- to post-diagnosis did not differ between women who developed cancer (breast or colorectal) and those who remained cancer-free [16], and the Danish Diet, Cancer and Health cohort, where women who were diagnosed with breast cancer also had a BMI change similar to those who remained cancer-free [17], although men in the same cohort who were diagnosed with cancer experienced a reduction in BMI relative to controls [18].

While these studies offer valuable insight into weight change following a cancer diagnosis, overall BMI changes may disguise differential patterns of change by weight status. Pre-diagnosis obesity could be associated with greater risk of weight increase if any underlying propensity exacerbated responses to the psychological stress of a cancer diagnosis, or amplified responses to pharmaceutical treatments that have a known risk of weight gain. Consistent with this, a recent study observed an association between obesity risk gene (*FTO*) status and weight gain in women diagnosed with breast cancer [19], although no control data were available to determine whether the same pattern was seen in normal ageing. Alternatively, a cancer diagnosis could act as a ‘teachable moment’ [20]; promoting healthy lifestyle changes and resulting in more effective weight control; one previous study found that patients with a higher BMI were at lower risk of post-diagnosis weight gain [19].

The present study was therefore designed to provide benchmark data on weight change in cancer survivors relative to cancer-free controls stratified by weight status. Using prospective data from two large population-based cohorts; one from the UK and one from the US, we examined the impact of a cancer diagnosis on BMI by pre-diagnosis weight status. Cancer-free participants from the same cohorts over the same time periods controlled for other causes of weight change.

## Methods

### Study populations and measures

The English Longitudinal Study of Ageing (ELSA) and the Health and Retirement Study (HRS) are longitudinal population-based studies of UK and US adults aged ≥50 years. They have a degree of harmonisation in their data collection protocols, and both record weight status and major health events. Details on the cohorts and sampling methods have been published elsewhere [21, 22], and participants gave full informed consent, with ethical approval obtained from the relevant bodies. ELSA data are publicly available at http://discover.ukdataservice.ac.uk and HRS data are available at https://ssl.isr.umich.edu/hrs/start.php.

#### English Longitudinal Study of Ageing

ELSA is a panel study recruited from households with one or more members aged ≥50 years responding to the Health Survey for England (HSE) in 1998, 1999, and 2001 (core sample: *N* =12099), with ‘refreshment samples’ added from additional rounds of the HSE in 2006, 2008, and 2012. They have been interviewed in biennial waves from 2002. At each wave, participants do a computer-assisted personal interview and complete self-administered questionnaires. In alternate waves a nurse visits the home to carry out a health examination that includes anthropometry. To date, three health examinations have been conducted; in 2004 (wave 2), 2008 (wave 4), and 2012 (wave 6). Anthropometric data from these waves were used for the present analyses, with information on cancer diagnoses taken from questionnaire data in waves 2–6.

#### Health and Retirement Study

HRS is a cohort study of US adults born between 1931 and 1941, plus their spouses or partners regardless of age (core sample: *N* =12652). Refreshment samples are added every three waves (six years). Participants are interviewed every two years, and the interviews include questions on new cancer diagnoses as well as self-reported anthropometric data. To match the time intervals (four years) for which nurse-measured anthropometric data were available for ELSA, we used anthropometric data from waves 2, 4, 6, 8, and 10 of HRS, and cancer diagnoses reported in waves 2–10.

Age, sex, and household non-pension wealth (a sensitive indicator of socioeconomic status in this age group) were included as covariates in all analyses.

### Cancer and comparison groups

The cancer survivor group in the ELSA cohort comprised all respondents who reported a new cancer diagnosis in waves 3 to 6. In the HRS cohort it comprised all respondents who reported a new cancer diagnosis in waves 3 to 10. A cancer diagnosis was defined as answering ‘yes’ to the question: ‘*Have you ever been told by a doctor or other health professional that you had cancer or any other kind of malignancy’.* Individuals in either cohort reporting a cancer diagnosis at waves 1 or 2 were excluded from the analysis because of the absence of pre-diagnosis BMI data. Likewise, participants from a refreshment cohort reporting a cancer diagnosis on joining the study were excluded for the same reason. The longer time period of data collection in HRS resulted in larger samples with BMI data over the two time points of cancer survival and controls.

Because the analyses involved BMI change, participants were only included if they had anthropometric data available both pre-and post-diagnosis. In ELSA, the post-diagnosis point was wave 4 for patients reporting a new diagnosis in waves 3 or 4, and wave 6 for patients reporting a new diagnosis in waves 5 or 6. The respective pre-diagnosis points were waves 2 and 4. In HRS we adopted a matched approach so that the post-diagnosis point was the first even-numbered wave at or after a new cancer diagnosis, and the previous even-numbered wave constituted the pre-diagnosis point.

In both samples, the comparison group comprised all individuals who had not received a cancer diagnosis in any wave and for whom full anthropometric data were available for the waves selected to match the pre- and post-diagnosis points. We selected all participants without a cancer diagnosis rather than a completely healthy control group because it enabled us to determine the specific additional influence of a cancer diagnosis independent of other chronic diseases. To match the ‘pre-diagnosis’ BMI, we used the mean of all possible pre-diagnosis waves (waves 2 and 4 in ELSA, and waves 2, 4, 6, and 8 in HRS). The matched ‘post-diagnosis’ BMI in the comparison sample was the mean of all possible post-diagnosis waves (waves 4 and 6 in ELSA, and waves 4, 6, 8, and 10 in HRS); giving an average interval of four years to match that of the cancer group’s pre- to post-diagnosis interval.

### Statistical analysis

Analyses were performed using SPSS version 20, with a *p* value < .05 determining statistical significance. Data were analysed separately for each cohort because participants were drawn from different populations, there were differences in measures (e.g. objectively measured vs. self-reported weight and height), and because it allowed us to replicate findings in two independent samples. We used repeated-measures analyses of variance (ANOVAs) in each cohort to first examine the group-by-time interaction (differential change in BMI between cancer and comparison groups), similar to other studies in the field that have not examined the effect of pre-diagnosis weight status. We then examined the three-way interaction between group (cancer vs. control), time (pre- vs. post-diagnosis), and pre-diagnosis weight status (normal weight: BMI <25 kg/m^2^, overweight: BMI 25–29.9 kg/m^2^, obese: BMI ≥30 kg/m^2^) to test the hypothesis that the BMI change would vary by weight status. All these analyses controlled for age, sex, and wealth at the pre-diagnosis time point. Because previous studies indicated potential sex differences in changes in BMI following a cancer diagnosis, we repeated analyses stratified by sex (controlling for age and wealth). We selected BMI, rather than weight, as our outcome variable for consistency with the previous literature, but we also ran all analyses on weight as a sensitivity check.

## Results

The analysed sample comprised participants who had data on height and weight on at least two consecutive even waves of data collection (four years apart), and were cancer-free at the first time. A new diagnosis of cancer during the study period (the ‘cancer survivor group’) occurred in 264 individuals in ELSA and 2553 in HRS. The comparison group comprised 1538 individuals in ELSA and 4946 in HRS who remained cancer-free. Cancer diagnoses were spread evenly across waves. In ELSA, 49% of the new diagnoses were at waves 3 or 4, and 51% at waves 5 or 6. In HRS, 25% of new diagnoses were at waves 3 or 4, 30% at waves 5 or 6, 22% at waves 7 or 8, and 23% at waves 9 or 10.

Baseline demographic and anthropometric characteristics of the cancer and comparison groups in ELSA and HRS are shown in Table 1. In both cohorts, the cancer survivors were older (*p* < .001) and included a higher proportion of men (ELSA *p* = .038, HRS *p* < .001) than the comparison group. The groups did not differ significantly by wealth in either cohort. The cancer survivors in both cohorts were taller (*p* < .001) and heavier (ELSA *p* = .035, HRS *p* < .001) than the comparison group, primarily due to the higher proportion of men. Mean BMI was significantly higher in the cancer survivors than the comparison group in ELSA (*p* < .001) but did not differ between groups in HRS. The cancer survivors in ELSA were more likely to be overweight or obese than the comparison group. The cancer survivors in HRS were less likely to be normal weight and more likely to be underweight.

**Table 1:** **Baseline characteristics of the cancer group and comparison group in the two cohorts – percentage (** ***n*** **), mean (SD)**

	ELSA cohort			HRS cohort		
	Cancer group	Comparison group	***p***	Cancer group	Comparison group	***p***
	(***n***= 264)	(***n***= 1538)		(***n***= 2553)	(***n***= 4946)	
**Demographic characteristics**						
Age (years)	66.33 (8.36)	63.47 (7.95)	<.001	67.24 (9.31)	64.08 (7.69)	<.001
Sex						
Male	51.1% (135)	38.6% (594)	<.001	54.2% (1385)	36.8% (1819)	<.001
Female	48.9% (129)	61.4% (944)	-	45.8% (1168)	59.6% (2949)	-
Wealth quintile						
1 (lowest)	14.3% (37)	12.7% (194)	.768	16.1% (412)	13.8% (660)	.070
2	17.8% (46)	18.8% (288)	-	18.3% (467)	19.3% (918)	-
3	21.3% (55)	20.3% (311)	-	21.8% (557)	21.2% (1009)	-
4	19.4% (50)	22.5% (344)	-	21.2% (542)	22.4% (1070)	-
5 (highest)	27.1% (70)	25.7% (394)	-	22.5% (575)	23.3% (1111)	-
Anthropometric characteristics*						
Weight (kg)	77.23 (14.64)	72.98 (15.04)	<.001	79.86 (17.06)	77.73 (16.40)	<.001
Height (cm)	166.44 (8.96)	165.17 (9.08)	.035	170.71 (10.00)	168.15 (9.70)	<.001
BMI (kg/m^2^)	27.87 (4.86)	26.71 (4.97)	<.001	27.34 (5.16)	27.39 (4.84)	.654
Weight status						
Underweight	0.8% (2)	1.0% (15)	<.001	1.6% (42)	0.5% (24)	<.001
Normal weight	28.4% (75)	45.6% (702)	-	32.6% (832)	33.5% (1658)	-
Overweight	42.0% (111)	31.3% (481)	-	40.7% (1038)	40.7% (2015)	-
Obese	28.8% (76)	22.1% (340)	-	25.1% (641)	25.3% (1249)	-

*Based on measured data in ELSA and self-reported in HRS.

Where percentage (*n*) is given numbers may not sum to the total sample number, as some items were not answered by all participants. Valid percentages are shown for ease of comparison between groups.

**Figure 1:**
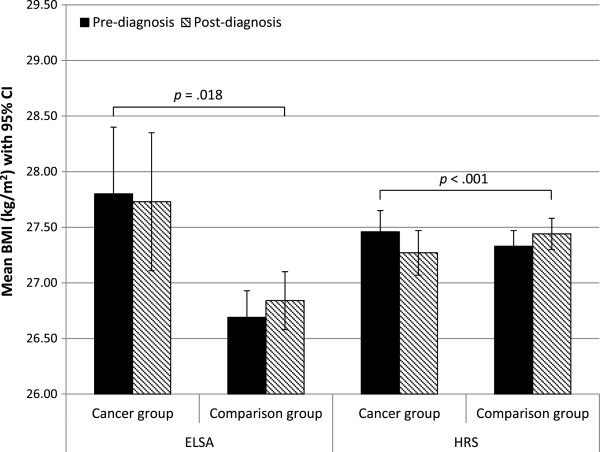
**Mean BMI at baseline and follow-up in the cancer group and the comparison group in the two cohorts.**

BMI decreased over time in the cancer survivors and increased in the comparison group. From pre- to post-diagnosis in ELSA, mean BMI change was -0.07 kg/m^2^ (SD = 2.22) in the cancer survivors and +0.14 kg/m^2^ (SD = 1.11) in the comparison group. In HRS, it was -0.20 kg/m^2^ (SD = 2.84) in the cancer survivors and +0.11 kg/m^2^ (SD = 0.93) in the comparison group. Figure 1 presents mean BMI values (adjusted for age, sex, and wealth) pre-diagnosis and post-diagnosis in the cancer survivors and the comparison group in each cohort. The group-by-time interaction, including the demographic covariates, was significant in ELSA (*p* = .018) and HRS (*p* < .001).

**Figure 2:**
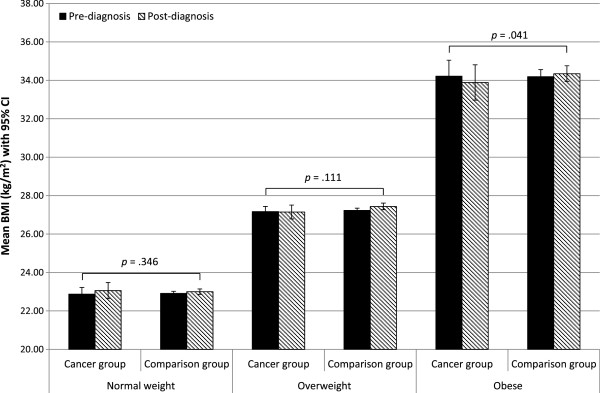
**Mean BMI at baseline and follow-up in the cancer group and the comparison group in the ELSA cohort by pre-diagnosis weight status.**

The three-way interaction between group, time, and pre-diagnosis weight status was significant in both cohorts (ELSA *p* = .015; HRS *p* < .001), with the cancer-control differences in BMI change being greatest among those who were obese pre-diagnosis. In ELSA, the mean BMI change in cancer survivors who had been normal weight pre-diagnosis was +0.19 kg/m^2^ (SD = 1.53), compared with -0.03 kg/m^2^ (SD = 1.99) in survivors who had been overweight, and -0.33 kg/m^2^ (SD =3.04) in those who had been obese. In the ELSA comparison group the respective figures were +0.09 kg/m^2^ (SD = 0.81), +0.20 kg/m^2^ (SD = 1.18), and +0.16 kg/m^2^ (SD = 1.50) (Figure 2).

**Figure 3:**
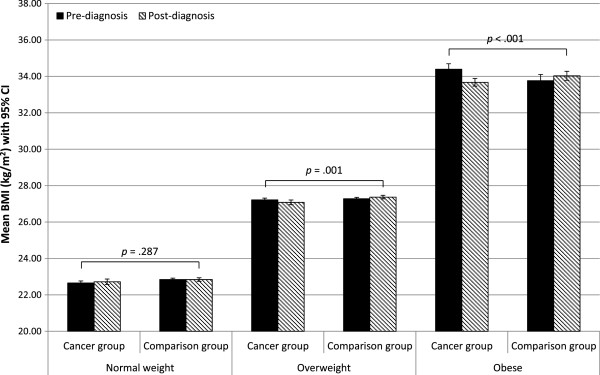
**Mean BMI at baseline and follow-up in the cancer group and the comparison group in the HRS cohort by pre-diagnosis weight status.**

In HRS, the mean BMI change in the cancer survivors who had been normal weight was +0.07 kg/m^2^ (SD = 2.30), compared with -0.14 kg/m^2^ (SD = 2.69) in survivors who had been overweight, and -0.72 kg/m^2^ (SD = 3.53) in those who had been obese. In the HRS comparison group the respective figures were +0.003 kg/m^2^ (SD = 0.66), +0.09 kg/m^2^ (SD = 0.85), and +0.27 kg/m^2^ (SD = 1.27) (Figure 3).

In analyses stratified by pre-diagnosis weight status, the group-by-time interaction was not statistically significant in normal weight participants in either cohort (ELSA *p* = .346, HRS *p* = .287). It was significant in overweight participants in HRS (*p* = .001) but not ELSA (*p* = .111), and was significant in obese participants in both cohorts (ELSA *p* = .041, HRS *p* < .001) (see Figures 2 and 3).

Sex-stratified analyses showed a significant three-way interaction between group, time, and pre-diagnosis weight status in women in ELSA (*p* = .021) and men and women in HRS (*p*s < .001), but the interaction did not reach significance in men in ELSA (*p* = .118). When we examined differences in BMI change over time between the cancer group and comparison group by sex and weight status (Additional file 1), we observed no significant group by time interaction in normal weight men or women in either ELSA (men *p* = .540; women *p* = .724) or HRS (men *p* = .493; women *p* = .462). Similarly, the group-by-time interaction was not significant in overweight men or women in ELSA (men *p* = .054; women *p* = .567) or overweight men in HRS (*p* = .103) – although in each group there was a trend towards greater weight loss among those who received a cancer diagnosis than those who did not – however, it was highly significant in HRS women (*p* < .001). Among obese participants, the group-by-time interaction was significant in women in ELSA (*p* = .013) and men and women in HRS (*p*s < .001), but was not significant in ELSA men (*p* = .557). We reran all analyses with weight as the outcome variable and observed no notable differences in the results.

## Discussion

This study used prospective data from population-based samples of older adults in the UK and the US to examine the effect of a cancer diagnosis on BMI in relation to pre-diagnosis weight status. In both samples, obese individuals who received a cancer diagnosis experienced a small but significant reduction in BMI from pre- to post-diagnosis, while there was little change in BMI in obese individuals who remained cancer-free. In contrast, among normal weight individuals in both samples, there was no differential BMI change related to a cancer diagnosis. Among the overweight, the pattern was similar to the obese (greater weight loss in those who got a cancer diagnosis) which was significant in HRS, but not significant in the smaller ELSA sample.

Two previous studies had found no significant differences between women who received a breast cancer diagnosis and those who remained cancer-free [16, 17], but they did not test the interaction with pre-diagnosis weight status. A third study that compared change in BMI among men diagnosed with any cancer with cancer-free controls found that a cancer diagnosis was associated with a significant reduction in BMI, but again did not examine differences by pre-diagnosis weight status [18]. In the present study, the pattern of results did not differ by sex in the HRS cohort, with significant differences between obese cancer cases and obese controls in BMI change over time, but no difference between normal weight groups. We saw the same pattern of results in women in ELSA, but found no significant differences in BMI change over time in any weight group in men in ELSA.

We did not have data on whether weight loss was intentional, but the fact that the reduction in BMI was not observed in cancer survivors with a healthy BMI, but was seen among those who had been obese pre-diagnosis, suggests that it may have been at least partly intentional. There have been few investigations of cancer survivors’ beliefs about weight loss, but a recent survey of 200 breast cancer survivors indicated widespread belief that weight loss is beneficial, with 70% believing that limiting food intake to maintain or lose weight could reduce the risk of recurrence [23]. Deliberate attempts to lose weight were also common in the breast cancer sample, with 65% having limited their intake during the last month to this end [23]. In another study, 87% of cancer survivors thought that advice on weight loss for cancer patients would be beneficial and the same number thought it was doctors’ duty to provide such advice [24]. That we saw a stronger impact of a cancer diagnosis on weight change in women than men in the ELSA cohort also points to weight loss being intentional, given that obese women tend to be more likely than obese men to recognise that they are too heavy [25], and more likely to report trying to lose weight [25].

However, an alternative explanation for the observed interaction with weight status is that obese cancer survivors had more advanced cancers than the normal weight survivors, and their greater weight loss was a consequence of this. Several studies suggest that obese individuals are less likely to participate in age-appropriate cancer screening programmes [26–29], and studies in breast cancer populations have identified obesity as a risk factor for patient delay (time from onset of first symptoms to first consultation of a doctor) [30], and advanced stage at diagnosis [31, 32]. Because weight loss is a common feature in advanced cancers, affecting between 39% and 82% of patients [33], if the obese cancer survivors in our sample had more advanced disease than the normal weight survivors, this could explain the differential BMI change.

The present findings showing weight loss occurring in individuals who receive a cancer diagnosis highlight the importance of future research to clarify whether weight loss is a deliberate health promoting activity or is a more ominous sign of underlying health state. Understanding the implications of weight loss among obese individuals who receive a cancer diagnosis is important for tailoring lifestyle advice and/or identifying those at higher risk of mortality. Future work not only needs information on cancer site, but also on disease stage.

The consequences of weight loss for cancer survivors is a crucial issue in survivorship research. Observational studies have demonstrated associations between weight loss and increased risk of recurrence and higher all-cause mortality in several large cohorts of breast cancer survivors [34–36], with similar adverse effects reported in smaller samples of colorectal and endometrial cancer survivors [37, 38]. In one study [34], associations between weight loss and mortality were stratified by weight status, and there was no evidence that weight loss was less harmful in the obese, although like the present study, there was no information on whether or not the weight loss was intentional. In non-cancer populations, unintentional weight loss is associated with mortality, whereas intentional weight loss has an overall neutral effect on survival [39]. Intervention trials offer better insight into the consequences of intentional weight loss, but no trials to date have directly investigated the effect on survival, although a study looking at the impact of weight loss on breast cancer recurrence and survival is underway [40]. However, comparison of the results of two large intervention studies of dietary change in breast cancer survivors (the Women’s Intervention Nutrition Study (WINS) [41] and the Women’s Healthy Eating and Lifestyle (WHEL) study [42]) suggests that diet-induced weight loss might have a favourable effect on recurrence. Both studies achieved positive changes in diet in the intervention group, but only WINS achieved significant weight loss, and only in WINS were recurrence rates lower in the intervention group. Further evidence for potential benefits of weight loss comes from small randomised controlled trials of overweight and obese breast cancer survivors which have examined cancer-related biomarkers. In the Breast Cancer Survivors Health and Physical Exercise (SHAPE) trial, postmenopausal survivors who lost at least 5% of their body weight had lower levels of oestrone, oestradiol, and bioavailable oestradiol than women who did not achieve the same weight loss [43]. In another trial there were favourable changes in sex hormone-binding globulin, leptin, high-sensitivity C-reactive protein, and total cholesterol in women who lost at least a kilogram in weight [44]. Given that these biomarkers have been associated with cancer recurrence and progression [45, 46], the results suggest that intentional weight loss may lead to improved outcomes in breast cancer survivors. However, evidence of poorer outcomes associated with weight loss in the larger observational studies, alongside modest evidence for improved outcomes with intentional weight loss, underscores the need for research into the determinants and consequences of weight loss following a cancer diagnosis.

The present study had some strengths. It is one of only a few studies to examine change in BMI from pre-diagnosis to post-diagnosis using a prospective design, thus minimising the potential for reporting bias. It also included cancer-free controls in order to distinguish changes related to a cancer diagnosis from those occurring naturally with age in the population. Finding the same pattern of results in two independent cohorts attests to the robustness of the effect. The availability of objective measurements of height and weight in ELSA is an advantage because all the previous controlled longitudinal studies have relied on self-reported data on at least one time point [16–18].

However, there were also a number of limitations. Cancer data were self-reported, but this may not be too problematic given previous studies have shown high agreement between self-reported cancer diagnoses and medical record validation in population-based samples [47–49]. We do not have information on the exact date of diagnosis, which could have been any time from just after the last wave at which the participant reported not having a cancer diagnosis, until just before the wave at which a cancer diagnosis was first reported; a range of two years. We also have no available data on stage at diagnosis, nor on weight loss intentions, and so it was not possible to test whether the interaction with weight status was a consequence of obese participants being more likely to be diagnosed at an advanced stage, or of making intentional efforts to reduce BMI [31, 32]. The study was not powered to analyse changes by cancer site, and given the substantial heterogeneity across cancers it is likely that results would differ by site. In order to study change in BMI over time, our analyses were limited to participants with data on at least two consecutive waves with nurse measurements available in ELSA (four years apart) and at the same intervals in HRS. Participants who died, dropped out, did not answer the cancer diagnosis question, or did not have data on BMI were therefore not included. The analysed samples were slightly younger and wealthier than the total ELSA and HRS samples, in line with retention in other longitudinal studies [50], so results may not be population-representative. In addition, the cancer group was necessarily restricted to those who were still alive at follow-up and sufficiently well enough to participate, so the results cannot be generalised to cases with more aggressive cancers.

## Conclusions

In conclusion, in large samples from two countries, we found that BMI decreased more following a cancer diagnosis in individuals who were obese beforehand than those who had been normal weight before diagnosis; but no such difference was observed over the same time period in cancer-free controls. With observational evidence suggesting that weight loss is associated with poorer outcomes for cancer survivors, but emerging trial evidence indicating there may be benefits of *intentional* weight loss for those who are overweight or obese, it is vital to get a better understanding of the determinants and consequences of weight loss following a cancer diagnosis to understand its full clinical implications. Clinical populations could extend the present findings by offering insight into differences in change in BMI by cancer site, stage at diagnosis, time since diagnosis, and treatment method.

## Electronic supplementary material

Additional file 1: **Mean (SD) changes in BMI (kg/m** ^**2**^ **) over time in the cancer group and comparison group in the two cohorts, and** ***p*** **values for the group by time interaction, by sex and weight status.** (DOCX 17 KB)

## Abbreviations

ELSA: English Longitudinal Study of Ageing
HRS: Health and Retirement Study
HSE: Health Survey for England
WINS: Women’s Intervention Nutrition Study
WHEL: Women’s Healthy Eating and Lifestyle study.
