# Supplementary material for: The impact of a cancer diagnosis on weight change: findings from prospective, population-based cohorts in the UK and the US
Source: BMC Cancer. 2014 Dec 9;14:926. doi: 10.1186/1471-2407-14-926 (PMC4265482; doi:10.1186/1471-2407-14-926)
Supplement: Supplementary file 1 — Additional file 1: Mean (SD) changes in BMI (kg/m 2 ) over time in the cancer group and comparison group in the two cohorts, and p values for the group by time interaction, by sex and weight status. (DOCX 17 KB) [file 12885_2014_5078_MOESM1_ESM.docx]

## Additional file 1

| **Additional file 1** Mean (SD) changes in BMI (kg/m^2^) over time in the cancer group and comparison group in the two cohorts, and *p* values for the group by time interaction, by sex and weight status | | | | | | | | |
| --- | --- | --- | --- | --- | --- | --- | --- | --- |
|  | | **ELSA cohort** | | | | | | |
|  | | **Men** | | |  | **Women** | | |
|  | | **Cancer group** | **Comparison group** | ***p*** |  | **Cancer group** | **Comparison group** | ***p*** |
| **Weight status** | |  |  |  |  |  |  |  |
|  | Normal weight | +0.17 (1.31) | +0.08 (0.69) | .540 |  | +0.15 (1.80) | +0.09 (0.87) | .724 |
|  | Overweight | -0.12 (1.44) | +0.18 (0.93) | .054 |  | +0.09 (2.61) | +0.23 (1.35) | .567 |
|  | Obese | +0.40 (1.53) | +0.21 (1.35) | .557 |  | -0.70 (3.54) | +0.13 (1.58) | .013 |
|  | | **HRS cohort** | | | | | | |
|  | | **Men** | | |  | **Women** | | |
|  | | **Cancer group** | **Comparison group** | ***p*** |  | **Cancer group** | **Comparison group** | ***p*** |
| **Weight status** | |  |  |  |  |  |  |  |
|  | Normal weight | +0.03 (2.32) | -0.05 (0.56) | .493 |  | +0.09 (2.27) | +0.03 (0.70) | .462 |
|  | Overweight | -0.10 (2.44) | +0.04 (0.73) | .103 |  | -0.22 (3.07) | +0.14 (0.93) | <.001 |
|  | Obese | -0.77 (3.19) | +0.28 (1.05) | <.001 |  | -0.68 (3.83) | +0.26 (1.38) | <.001 |
| All values are adjusted for age and wealth. | | | | | | | | |
